# HPV associated oral lesions among HIV/AIDS patients - a cross sectional study

**DOI:** 10.1186/1471-2334-14-S3-E31

**Published:** 2014-05-27

**Authors:** T Smitha, CV Mohan

**Affiliations:** 1 Department of Oral Pathology, V.S.D.C.H, Bangalore, India; 2 Dental Care and Research Centre, Basavanagudi, Bangalore, India

## Background

Oral HPV infection results from increase in oral based immune suppression in the HIV co infected individual that cannot be restored and is possibly compromised further by HAART. Unfortunately, little is known about the prevalence and immune response against oral HPV infection.The aim of the present study was to study the prevalence of HPV associated lesions in a group of patients with HIV who were on HAART.

## Methods

Cross sectional, descriptive and observational study was carried out among HIV/AIDS patients attending the ART centre, KIMS, Bangalore from January 2010 to June 2012. Previous informed consent was taken and after which a detailed oral exam, was carried out in patients considering WHO diagnostic criteria. Demographic and clinical profile of HPV associated oral lesions was recorded according to the WHO diagnostic criteria.

## Results

Among 318 patients with 540 lesions, majority of the lesions belonged to one of the five categories of ulcers (13.3%), warts (3%), candidiasis (36.45%), pigmented and white lesions (47.2%). Among the warty lesions screened the most common lesion was verruca vulgaris (8 cases), followed by papilloma (4 cases) and focal epithelial hyperplasia (1 case). Oral SCC was detected in two patients and they were confirmed by histopathology. The warts biopsied showed characteristic papilloma features with two cases showing dysplastic changes.

## Conclusion

There was a large reduction in the presentation of oral ulcers, and there appeared to be a relative increase in papillomatous lesions and a decrease of other mucosal diseases over the period of study.

